# Acrolein: blocking antibody formation: pro tumor, anti-allergy

**DOI:** 10.1186/2045-7022-4-S2-P31

**Published:** 2014-03-17

**Authors:** Franziska Roth-Walter, Anna Willensdorfer, Caroline Stremnitzer, Cornelia Schultz, Susanne Diesner, Krisztina Szalai, Judit Fazekas, Anna Moskovskich, Alina Neunkirchner, Hanna Birnleitner, Erika Jensen-Jarolim, Erika Jensen-Jarolim

**Affiliations:** 1 University of Veterinary Medicine, Messerli Research Institute, Comparative Medicine, Vienna, Austria; 2 Medical University of Vienna, Department of Pathophysiology and Allergy Research, Vienna, Austria; 3 Medical University of Vienna, Department of Pediatrics and Adolescent Medicine, Vienna, Austria; 4 Medical University of Vienna, Institute of Immunology, Vienna, Austria

## Background

Allergic sensitization has been linked to active and passive smoking in exposed individuals and even their pets. We here investigated the contribution of acrolein, a compound generated in large amounts during smoking, during nasal sensitization and -- based on the surprising preliminary results -- on tumor growth. As a model antigen we used KLH with or without acrolein.

## Methods

BALB/c mice were nasally sensitized 5 times in biweekly intervals with KLH alone or with KLH in conjunction with acrolein. Airway hyperreactivity was was measured according to change of enhanced pause and KLH-specific anaphylactic reaction was monitored in vivo Levels of specific antibodies as well as cytokine profile of KLH-stimulated splenocytes were analyzed by ELISA. Further, mouse D2F2-tumor cells were grafted to the flanks and tumor growth monitored in mice previously exposed to acrolein or buffer.

## Results

Nasal application of KLH as model antigen induced specific IgG1-, IgG2a-, IgA- and IgE-levels. The same mice secreted elevated levels of IL5, IL13, IL10 and IFN-γ. They showed increased airway-hyperreactivity and had a significant drop in body temperature upon allergen challenge. Pointing towards tolerance, and against our expectations, presence of acrolein in the KLH-antigen significantly reduced specific antibody-titers, resulted in lower splenocyte cytokine production and prevented anaphylaxis. However, the impaired immune response simultaneously led to a significantly higher tumor growth in mice exposed to acrolein than in the control group.

## Conclusion

Acrolein in smoke -- best known for its carcinogenic effect - decreases the risk of sensitization towards a specific antigen by inhibiting immune activation. Our data further suggest that Acrolein via the same mechanism acts tumor promoting in smokers.

